# Comparative Study of Metal Substrates for Improved Carbonization of Electrospun PAN Nanofibers

**DOI:** 10.3390/polym14040721

**Published:** 2022-02-13

**Authors:** Jan Lukas Storck, Martin Wortmann, Bennet Brockhagen, Natalie Frese, Elise Diestelhorst, Timo Grothe, Christian Hellert, Andrea Ehrmann

**Affiliations:** 1 Faculty of Engineering and Mathematics, Bielefeld University of Applied Sciences, 33619 Bielefeld, Germany; jan_lukas.storck@fh-bielefeld.de (J.L.S.); bennet.brockhagen@fh-bielefeld.de (B.B.); elise.diestelhorst@fh-bielefeld.de (E.D.); timo.grothe@fh-bielefeld.de (T.G.); christian.hellert@fh-bielefeld.de (C.H.); 2 Faculty of Physics, Bielefeld University, 33615 Bielefeld, Germany; martin.wortmann@fh-bielefeld.de (M.W.); nfrese@uni-bielefeld.de (N.F.)

**Keywords:** electrospinning, poly(acrylonitrile), stabilization, carbonization, metallic substrates, shrinkage, nanofiber morphology

## Abstract

Carbon nanofibers are used for a broad range of applications, from nano-composites to energy storage devices. They are typically produced from electrospun poly(acrylonitrile) nanofibers by thermal stabilization and carbonization. The nanofiber mats are usually placed freely movable in an oven, which leads to relaxation of internal stress within the nanofibers, making them thicker and shorter. To preserve their pristine morphology they can be mechanically fixated, which may cause the nanofibers to break. In a previous study, we demonstrated that sandwiching the nanofiber mats between metal sheets retained their morphology during stabilization and incipient carbonization at 500 °C. Here, we present a comparative study of stainless steel, titanium, copper and silicon substrate sandwiches at carbonization temperatures of 500 °C, 800 °C and 1200 °C. Helium ion microscopy revealed that all metals mostly eliminated nanofiber deformation, whereas silicone achieved the best results in this regard. The highest temperatures for which the metals were shown to be applicable were 500 °C for silicon, 800 °C for stainless steel and copper, and 1200 °C for titanium. Fourier transform infrared and Raman spectroscopy revealed a higher degree of carbonization and increased crystallinity for higher temperatures, which was shown to depend on the substrate material.

## 1. Introduction

**Figure 1:**
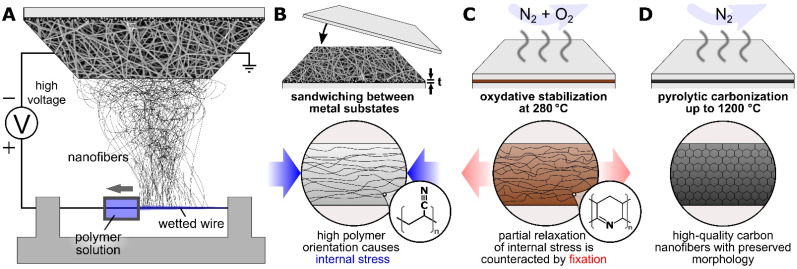
Schematic illustration of (**A**) wire-based electrospinning, (**B**) sample preparation, (**C**) stabilization and (**D**) carbonization using different metal substrates.

Poly(acrylonitrile) (PAN) nanofibers are typically produced either by needle-based or by needleless electrospinning methods (cf. Figure 1A) and are a common precursor for carbon nanofibers (CNF) [1,2]. Due to their outstanding electrical and mechanical properties, CNF are particularly promising for nano composites [3,4,5]. In particular, freestanding CNF mats with sufficient mechanical strength that can be used without a substrate have attracted considerable attention [6,7,8].

For the production of CNF, pristine PAN nanofibers are usually first oxidatively stabilized below 300 °C and then carbonized above 500 °C in an inert atmosphere. Much research has focused on the optimization of process parameters such as heating rate and terminal temperature [9,10,11]. Higher temperatures generally result in a higher degree of carbonization and crystallinity, thus improving stability and electrochemical properties [12,13]. The heating rate, especially during stabilization, plays a crucial role in retaining the original nanofiber morphology [2,12,13].

Another aspect, not related to thermal but to mechanical treatment, is the possible fixation or even stretching of pristine nanofibers during stabilization and carbonization. The thermally induced relaxation of internal stress, as introduced by extreme polymer chain elongation during electrospinning, usually results in undesirable deformation and contraction of the CNF, observed both macroscopically and on a nanometer scale [14,15,16]. This process can be counteracted by applying a force during the temperature treatment, either by fixation of the pristine nanofiber mat or by stretching of aligned nanofiber bundles [17,18,19]. While the latter method has been shown to work well in the literature, the former, although much simpler, can cause the nanofiber mat to break.

In an earlier study, we demonstrated that metallic substrates are a simple and efficient way to retain both the macroscopic dimensions of PAN-based CNF mats and the nanoscopic fiber morphology during the thermo-oxidative stabilization. For this, the pristine PAN nanofibers are spun directly onto aluminum foil or other rigid metal substrates. The adhesion of the nanofibers to the substrate enables a uniform stress distribution preventing fiber deformation without breaking [20,21,22]. In our most recent study, we showed that stabilization and incipient carbonization could be improved by sandwiching the PAN nanofiber mats between aluminum or stainless steel sheets [23]. Aluminum, however, cannot be used for higher temperatures and is thus not suitable for high-temperature carbonization.

In this study, we compare different substrate materials that form sandwiches with the nanofiber mats (cf. Figure 1B) for incipient carbonization at 500 °C and, as far as possible with the chosen substrates, higher temperatures of up to 800 °C and 1200 °C (cf. Figure 1C,D), showing that different substrate materials are optimal for different carbonization temperatures. In this regard carbonization at 800 °C with copper substrates results in an ideal compromise of intact nanofibers and crystallinity, while titanium substrates enabled carbonization at 1200 °C.

## 2. Materials and Methods

PAN nanofiber mats were produced in a needleless electrospinning instrument Nanospider Lab (Elmarco, Liberec, Czech Republic). The spinning solution was prepared from 16% PAN (X-PAN, from Dralon, Dormagen, Germany), dissolved in dimethyl sulfoxide (DMSO, min. 99.9%; S3 Chemicals, Bad Oeynhausen, Germany), by stirring for 2 h at ambient temperature.

The spinning parameters were as follows: high voltage 80 kV, resulting current ~0.1 mA, nozzle diameter 0.9 mm, electrode–substrate distance 240 mm, carriage speed 100 mm/s, substrate speed 0 mm/min, relative humidity 32%, temperature in the spinning chamber 22 °C, and spinning duration 30 min. These parameters are identical to those chosen in the previous studies [21,22,23].

For comparison with the previous study [23], a stainless steel 1.4301 V2a sheet (thickness 500 µm; Stahlog GmbH, Hörselberg-Hainich, Germany) was again used to prepare substrate–nanofibers–substrate sandwiches for stabilization and subsequent carbonization. In addition, three substrates (used in [22] as single-sided supports, i.e., without capping the nanofiber mats by a second substrate) were investigated as sandwiches: copper foil, thickness 100 µm (Blechmaennle-de, Rottenburg, Germany); a titanium sheet, thickness 100 µm (Evek GmbH, Mühlheim an der Ruhr, Germany); and a silicon (100) wafer with an oxidized surface, thickness 525 µm (Science Service GmbH, Munich, Germany).

For stabilization, a muffle oven B150 (Nabertherm, Lilienthal, Germany) was used to keep the samples at a temperature of 280 °C for 1 h, approached with a heating rate of 0.25 K/min. Carbonization was carried out in a tube furnace CTF 12/TZF 12 (Carbolite Gero Ltd., Sheffield, UK) at 500 °C, 800 °C or 1200 °C for 1 h, all temperatures approached with heating rates of 10 K/min in a nitrogen gas flow of 100 mL/min (STP). Heating rates and stabilization temperature correspond to the values from our previous studies [21,22,23].

To examine the morphology of the nanofibers after stabilization and carbonization, a helium ion microscope (HIM) Orion Plus (Carl Zeiss, Jena, Germany) was used with 35.9 kV acceleration voltage. The spot control was defined as 6.5, resulting in a beam current of 0.6–0.7 pA. An electron flood gun was used to avoid charging effects during secondary electron detection.

ImageJ (version 1.53e, 2021, National Institutes of Health, Bethesda, MD, USA) was used to measure nanofiber diameters (from HIM images with field of view 8 µm, taking 100 measurements per images) and to count broken nanofiber ends (from HIM images with field of view 30 µm, using the Multi-point counting function).

For the investigation of the carbonization process, a Fourier-transform infrared (FTIR) spectrometer Excalibur 3100 (Varian Inc., Palo Alto, CA, USA) in attenuated total reflection mode (ATR-FTIR) was used in the wavenumber range from 4000–700 cm^−1^. For Raman investigations, a LabRAM Aramis spectrometer (HORIBA Europe, Oberursel, Germany) with a cooled CCD detector and a helium-neon laser at 633 nm was used in backscattering mode. The $I_{D}/I_{G}$ ratio was calculated from the peak amplitudes.

Sample thickness measurements were performed by a Fischerscope MMS PC2 with EGAB 1.3 probe (Helmut Fischer GmbH, Sindelfingen, Germany).

## 3. Results and Discussion

**Figure 2:**
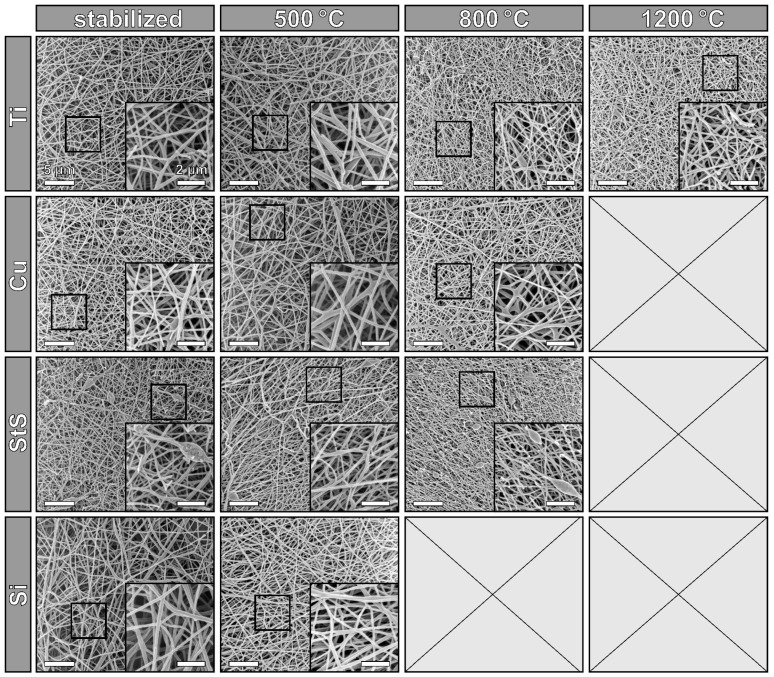
HIM images of CNF mats, stabilized and carbonized at different temperatures, sandwiched between titanium (Ti), copper (Cu), stainless steel (StS) sheets and silicon (Si) wafers, respectively. Scale bars define 5 µm in the large images and 2 µm in the insets.

**Figure 3:**
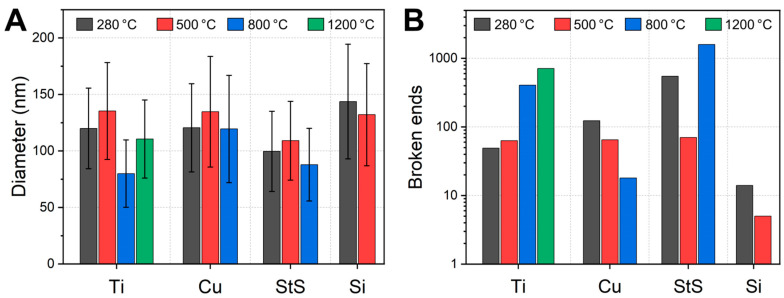
(**A**) Average nanofiber diameters and (**B**) numbers of broken nanofiber ends counted in the HIM images with a field of view of 30 × 30 µm^2^, as shown in Figure 2. The y-scale is logarithmic. A temperature of 280 °C refers to the stabilized samples.

For an overview of the nanofiber morphologies after stabilization and carbonization at different temperatures, Figure 2 depicts HIM images of all nanofiber mats. The open positions indicate samples, which were vanished after carbonization at the respective temperatures. Generally, all images show apparently intact nanofiber mats with random nanofiber distribution, as it is expected from needleless electrospinning with stationary substrates. The beads which are visible in a few images often occur when PAN is electrospun from DMSO, varying with small changes in the relative humidity in the spinning chamber. However, the diameter distributions vary slightly between the different substrates, as Figure 3A shows.

Comparing the diameters, no significant differences are visible. The values measured here are slightly smaller than those reported in [23] for incipient carbonization at 500 °C in aluminum sandwiches. There is neither a trend towards larger diameters for carbonized nanofibers, as could be expected if the nanofibers had shrunken, nor a trend towards smaller diameters after thermal treatment due to a significant mass loss. The diameter distributions, as indicated by the error bars, do not differ significantly, either. This result shows that all substrate materials used are well suited to preserve the nanoscopic fiber morphology in terms of diameter, topography and homogeneity during heat treatment.

The fixation of the nanofibers to the substrate surface generates mechanical tensions during heat treatment, which eventually oppose the deformation. It has already been shown [22,23] that this can lead to breakage of individual CNFs. Figure 3B thus shows the number of broken nanofiber ends, as counted in the HIM images shown in Figure 2. In a perfect CNF mat, nearly no broken nanofiber ends are visible since electrospun nanofibers typically have lengths in the range up to millimeters, making the probability of seeing fiber ends in images of only 30 µm edge length (cf. Figure 2) very low.

There is no general trend visible regarding the increase or decrease of the number of broken nanofibers with increasing treatment temperature. For the titanium substrates, the broken ends clearly increase with higher carbonization temperatures; however, the opposite trend is visible for the copper substrates. This is presumably due to the statistical variance between different sample regions in connection with the limited size of the HIM images, thus no generalizing conclusions can be drawn here [24].

Overall, the highest numbers of broken ends are reached with the stainless steel substrate, while the Si wafer seems to be, in this regard, ideally suited for stabilization and incipient carbonization at 500 °C.

**Figure 4:**
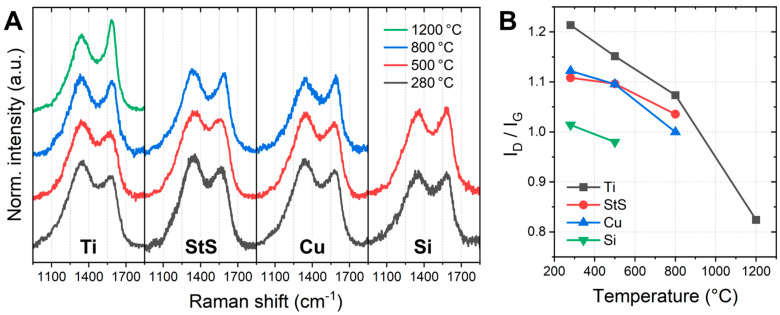
Raman investigations of the stabilized and carbonized nanofibers: (**A**) D and G band regions and (**B**) corresponding amplitude ratios *I_D_*/*I_G_*.

**Figure 5:**
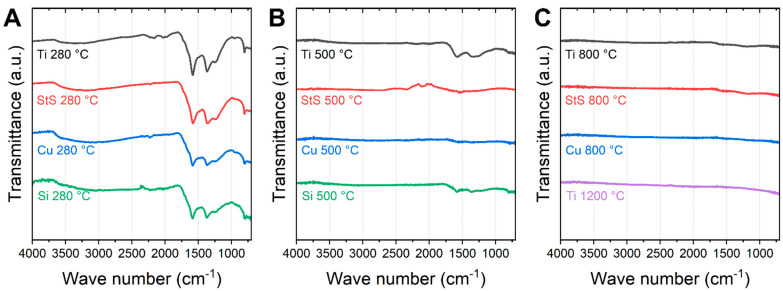
FTIR investigations of the stabilized and carbonized nanofibers: (**A**) stabilization on different substrates, (**B**) carbonization at 500 °C, and (**C**) carbonized at higher temperatures.

Besides the morphology, the degrees of carbonization and crystallinity are crucial determinants of the mechanical and electrical properties of the CNF obtained. Figure 4 shows Raman spectra in the D band (around 1350 cm^−1^) and G band (around 1580 cm^−1^) regions of the stabilized and carbonized nanofiber mats. While the G band is related to in-plane bond-stretching motion of pairs of sp^2^ hybridized carbon atoms the D band stems from defects in the aromatic ring structure of the graphite lattice or domain boundaries of graphite crystallites [25,26]. The ratio *I_D_*/*I_G_* thus gives the ratio of disordered to ordered graphitic domains, i.e., a measure for crystallinity of the samples, with higher crystallinity being indicated by a lower ratio *I_D_*/*I_G_* [27]. Interestingly, Figure 5 shows not only increasing crystallinity for increasing carbonization temperature, but also differences between the substrate materials. While the highest crystallinity is reached for carbonization at 1200 °C using Ti substrates, similar values were also reached at lower temperatures of 800 °C for the Cu substrate and even for 500 °C for the Si substrate. Measuring *I_D_*/*I_G_* after incipient carbonization at 500 °C in an aluminum sandwich resulted in a value of approx. 1.2 [23], i.e., a lower crystallinity than found for the materials used in the recent study. It can be concluded that the metal substrate, possibly through catalytic activity in the pyrolysis reaction, has a significant effect on the degree of crystallization of the CNF, which may allow similar results to be obtained at much lower temperature via substrate variation.

To investigate the chemical properties, i.e., the gradual removal of oxygen-functional groups, FTIR spectroscopy was performed. The results are depicted in Figure 5. After stabilization, the curves measured on different substrates are mostly similar. The well-known peaks occur around 800 cm^−1^ (aromatic C−H ring bending vibrations), 1575 cm^−1^ (C=N and C=C stretching vibrations), 1370 cm^−1^ (C–H deformation), and 1240 cm^−1^ (C–O vibrations due to oxygen crosslinking between the polymer chains) [9,28].

Pure carbon shows no peaks at all as it is almost chemically inert [29,30]. As can be seen in Figure 5B, only CNF carbonized on Ti and Si substrates show signs of residual functional groups after carbonization at 500 °C (the artifact near 2100 cm^−1^ visible for stainless steel stems from the incompletely compensated strong absorption of the diamond ATR crystal). In particular, CNF carbonized on Ti show pronounced bands between 1700 and 1000 cm^−1^, which is consistent with the lowest degree of crystallinity, as determined by Raman spectroscopy. Similarly, incipient carbonization at 500 °C in an aluminum sandwich resulted in nearly completely vanished peaks in the previous study [23]. At higher carbonization temperatures no distinct bands were observed (Figure 5C).

It should be mentioned that besides all aforementioned spinning, stabilization and carbonization parameters, the thickness of the specimen has a significant impact on the carbonization results. It has been observed that a higher sample thickness, i.e., spinning duration (cf. Figure 1B), stabilizes the CNF mats against macroscopic deformation and breaking. In this regard a (3.5 ± 0.8) µm thick nanofiber mat carbonized at 1200 °C on a Ti substrate had mostly maintained its macroscopic appearance (cf. Figure S1A in the supplementary materials), while a sample with a thickness of (1.1 ± 0.4) µm had shrunk significantly more (cf. Figure S1B in the supplementary materials). An even thinner specimen with (0.2 ± 0.1) µm completely folded in on itself and had no resemblance to the pristine nanofiber mat whatsoever. Apparently, a certain minimum thickness is necessary to maintain useful CNF after carbonization under these conditions.

## 4. Conclusions

PAN nanofiber mats were stabilized and subsequently carbonized at 500 °C, 800 °C and 1200 °C, sandwiched between different metal and metalloid substrates. For higher temperatures, a higher degree of carbonization and crystallinity was found. The highest nanofiber integrity was found for the Si substrate after carbonization at 500 °C, which was not sufficient to reach a high degree of carbonization, while such nanofibers may be well suitable for producing nano-composites with enhanced mechanical properties, as compared to the pure polymer. The Ti substrate enabled carbonization at 1200 °C, but showed lower degrees of carbonization than the other substrate materials at lower temperatures. While a high crystallinity was only achieved with the Ti substrate after carbonization at 1200 °C, Cu and StS substrates achieved a high degree of carbonization at only 500 °C. Since CNF from Cu substrates showed relatively small numbers of broken ends, all in all carbonization in a Cu sandwich at 800 °C offers a balanced optimum of carbonization, crystallinity, and intact nanofibers. The improvement of the carbonization process with regard to the resulting crystallinity and morphology is of general significance to any kind of application that has been proposed over recent years. Examples include the use of CNF in energy storage applications with regard to electrochemical properties or the application in composite materials that benefit from improved morphological and mechanical properties.

The results demonstrate that carbonization of PAN nanofibers on metal substrates has significant advantages over conventional methods, not only avoiding undesired nanofiber deformation but also enhancing the resulting CNF in terms of physiochemical properties.

## Data Availability

All data produced in this study are presented in this paper.

## Supplementary Materials

The following supporting information can be downloaded at: https://www.mdpi.com/article/10.3390/polym14040721/s1, Figure S1: Photographic images of specimens after carbonization in Ti sandwiches at 1200 °C for 1 h with different original thicknesses.
