# Supplementary material for: Comparative Study of Metal Substrates for Improved Carbonization of Electrospun PAN Nanofibers
Source: Polymers (Basel). 2022 Feb 13;14(4):721. doi: 10.3390/polym14040721 (PMC8877959; doi:10.3390/polym14040721)
Supplement: Supplementary file 1 [file polymers-14-00721-s001.zip › polymers-1590179-supplementary.pdf]

Supplementary Material

Comparative Study of Metal Substrates for Improved Carbonization of Electrospun PAN Nanofibers

Jan Lukas Storck [1], Martin Wortmann [2], Bennet Brockhagen [1], Natalie Frese [2], Elise Diestelhorst [1], Timo Grothe [1] Christian Hellert [1] and Andrea Ehrmann [1,*]

[1] Faculty of Engineering and Mathematics, Bielefeld University of Applied Sciences, 33619 Bielefeld, Germany; jan_lukas.storck@fh-bielefeld.de (J.L.S.); bennet.brockhagen@fh-bielefeld.de (B.B.); elise.diestelhorst@fh-bielefeld.de (E.D.); timo.grothe@fh-bielefeld.de (T.G.); christian.hellert@fh-bielefeld.de (C.H.)

[2] Faculty of Physics, Bielefeld University, 33615 Bielefeld, Germany; martin.wortmann@fh-bielefeld.de (M.W.); nfrese@uni-bielefeld.de (N.F.)

* Correspondence: andrea.ehrmann@fh-bielefeld.de

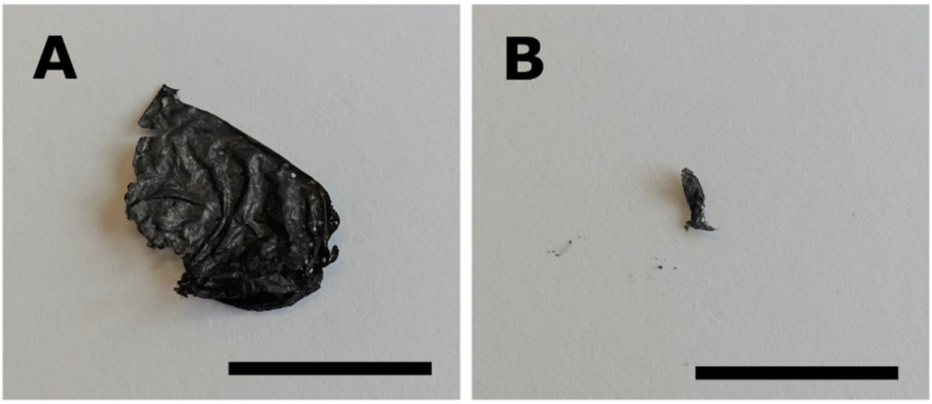

Figure S1. Photographic images of specimens after carbonization in Ti sandwiches at 1200 °C for 1 h with different original thicknesses: (**A**) 3.5 μm ± 0.8 μm and (**B**) 1.1 μm ± 0.4 μm. Scale bars depict 10 mm.
